# Development of electronic nose for detection of micro-mechanical damages in strawberries

**DOI:** 10.3389/fnut.2023.1222988

**Published:** 2023-07-31

**Authors:** Yingdong Qin, Wenshen Jia, Xu Sun, Haolin LV

**Affiliations:** ^1^Institute of Quality Standard and Testing Technology, Beijing Academy of Agriculture and Forestry Sciences, Beijing, China; ^2^College of Computer and Information Engineering, Beijing University of Agriculture, Beijing, China; ^3^Department of Risk Assessment Lab for Agro-products (Beijing), Ministry of Agriculture and Rural Affairs, Beijing, China; ^4^Key Laboratory of Urban Agriculture (North China), Ministry of Agriculture and Rural Affairs, Beijing, China; ^5^Lu'an Branch, Anhui Institute of Innovation for Industrial Technology, Lu'an, China; ^6^School of Mechanical Engineering and Automation, Liaoning University of Technology, Jinzhou, Liaoning, China; ^7^College of Computer and Information, China Three Gorges University, Yichang, China

**Keywords:** electronic nose, strawberry, mechanical damage, non-destructive testing, food inspection, embedded systems, classification model

## Abstract

A self-developed portable electronic nose and its classification model were designed to detect and differentiate minor mechanical damage to strawberries. The electronic nose utilises four metal oxide sensors and four electrochemical sensors specifically calibrated for strawberry detection. The selected strawberries were subjected to simulated damage using an H2Q-C air bath oscillator at varying speeds and then stored at 4°C to mimic real-life mechanical damage scenarios. Multiple feature extraction methods have been proposed and combined with Principal Component Analysis (PCA) dimensionality reduction for comparative modelling. Following validation with various models such as SVM, KNN, LDA, naive Bayes, and subspace ensemble, the Grid Search-optimised SVM (GS-SVM) method achieved the highest classification accuracy of 0.84 for assessing the degree of strawberry damage. Additionally, the Feature Extraction ensemble classifier achieved the highest classification accuracy (0.89 in determining the time interval of strawberry damage). This experiment demonstrated the feasibility of the self-developed electronic nose for detecting minor mechanical damage in strawberries.

## 1. Introduction

### 1.1. Background

Strawberries are popular small fruits that are well-suited for their delicious taste and rich nutritional value (1). Phenolic compounds in strawberries are known for their antioxidant and anti-inflammatory actions (2). They are abundant in vitamin C, anthocyanins, phenolic compounds, and other antioxidants (3), which promote antioxidation in the human body and reduce the risk of cardiovascular diseases and cancer (4). Strawberries also possess direct and indirect antimicrobial, anti-allergic, and antihypertensive properties (5). Although dried strawberries undergo dehydration, they retain high pro-health potential and maintain acceptable sensory qualities (6). Consequently, strawberries have become an indispensable part of their daily diet. Strawberries have various shapes and sizes, bright colours, smooth skin, tender and jurious flesh, and a rich taste. Conversely, strawberries have delicate exteriors, making them prone to mechanical damage during processes such as harvesting, transportation, and packaging (7). This minor damage may have accelerated the oxidation and deterioration of the strawberries. Moreover, strawberries are susceptible to fungal infections (8), and damaged areas can facilitate infection, leading to a decline in quality and waste from multiple perspectives. To avoid this situation, non-destructive detection and screening of strawberries with minor damage have become an important issue (9).

Electronic nose technology is an embodiment of the biomimetic olfaction concept that aims to mimic animal olfaction for fuzzy odour judgment without the need for precise chemical analysis. With the application of the e-nose technology, fruits can be tested in large quantities without damage. Detecting minor fruit damage, particularly during the early stages, can be challenging. In addition, image recognition methods may prove ineffective when fruits are stacked and obstruct each other. Traditional chemical analysis equipment typically requires sample extraction, which damages the external appearance of the fruit. This not only results in incomplete detection but also risks harming the packaging or fruit itself. In contrast, the electronic nose technology allows for rapid, non-destructive, and bulk assessments.

### 1.2. Research purpose and method

To ensure that the appearance and experience of strawberries are not negatively affected during sales, it is crucial to determine the timing of the damage and infer the cause in order to improve logistics and other pre-sales processes. Precise identification of damaged strawberry samples is needed so that they can be assessed and removed, providing a rapid, convenient, and non-destructive solution for the early detection of minor strawberry damage. We need to develop an electronic nose specifically designed to detect volatile gases emitted by strawberries using a sensor array that primarily meets the requirements for strawberry detection.

## 2. Literature review

### 2.1. Development of electronic nose for fruit and vegetable food inspection

In the 1960s, researchers designed microelectrodes to mimic the olfactory mechanism of animals by comparing it with that of olfactory hair. In 1963, Wilkens from Cornell University proposed that a differential response sensitivity to various volatiles could be obtained under any given microelectrode condition. By comparing the reactions to several odours under different electrode conditions, a differential response specificity similar to that of the different human olfactory receptor sites was discovered (10). In 1982, Persaud compared the olfactory system to an electronic nose. A comparison of electrical signals from sheep olfactory mucosa and semiconductor sensors showed some similarities. By adjusting these parameters, highly specific receptors can be replaced with a single sensor (11).

Electronic noses are widely used for the detection of diseases in fruits and vegetables. Gómez et al. used a portable PEN2 electronic nose device with ten different metal oxide sensors to evaluate the ability of electronic noses to monitor changes in volatile production during tomato ripening. Principal Component Analysis (PCA) and Linear Discriminant Analysis (LDA) were used to determine whether the electronic nose could distinguish different ripening stages (unripe, half-ripe, fully ripe, and overripe) (12). Chen et al. developed an electronic nose system for fruit ripeness and quality detection with an identification accuracy of up to 100% (13). Qian et al. detected Chinese noodles using an Electronic Nose (e-nose). The e-nose results indicated that the primary flavour differences in noodles were primarily attributed to inorganic sulphides, alcohols, aldehydes, and ketones (14). Zhao et al. proposed a rapid electronic nose method to detect whether apples were infected with fungi, and the recognition accuracy of the SSA-BPNN model reached 98.40% (15).

Commercial electronic noses commonly used for agriproduct detection include Germany’s AIRSENSE PEN 3 and France’s Alpha MOS Fox 4,000. In a study on the TVB-N content of eggs, Liu et al. used a Pen 3 electronic nose. Using the array response of the electronic nose, they established a content model capable of describing the egg storage period (16). Labreche et al. used a Fox 4,000 electronic nose to determine the shelf life of milk. An electronic nose was used to analyse milk samples stored at different temperatures and times (17).

### 2.2. The electronic nose as a common device for food detection

The electronic nose has always been a research hotspot for food detection. Elizabeth reviewed the applications of modern electronic noses and tongues in the food and pharmaceutical industries. The review covers various types of electronic nose sensors based on different principles, including organic polymers, metal oxides, quartz crystal microbalances, and the combination of gas chromatography (GC) and mass spectrometry (MS) (18). Shi et al. reviewed the development of electronic noses in science and technology, and their applications in fresh food. This review focuses on the sensing and recognition systems of electronic noses as well as their applications in fresh food classification, flavour detection, and spoilage evaluation (19). Sanaeifar et al. reviewed the applications of electronic noses in the food industry and discussed future development trends, prospects, and challenges (20). Jia summarised the research on agriproduct detection. This review discusses the applications of electronic noses in agriproduct analysis (such as fruits, vegetables, tea, grains, livestock meat, and fish), including freshness evaluation, quality classification, authenticity assessment, variety identification, geographical origin identification, and disease detection (21). Hotel reviewed algorithms for volatile compound recognition in electronic noses based on surface acoustic wave (SAW) sensors. This review describes several machine learning algorithms and compares their performance on different features used in state-of-the-art electronic nose systems (22). Zheng et al. reviewed electronic noses based on metal oxide sensors for crop pest detection. When crops are attacked by pests, they release (VOCs) to alert their natural enemies, which can then be captured using metal oxide semiconductor gas sensors. This review introduces the principles, techniques, and progress of crop pest detection (23). Baietto et al. summarised the applications of electronic noses in fruit identification, ripeness, and quality grading. This study reviews the chemical properties of fruit volatiles during fruit production, describes some more important applications provided by electronic nose (e-nose) technology for fruit aroma characterisation, and summarises recent research on e-nose data (24).

### 2.3. Study on strawberry-related gases, damage, and electronic nose detection

In 2006, Iannetta et al. investigated the relationship between ethylene (C2H4) and carbon dioxide (CO2) to represent strawberry ripening. Their experiments showed that the C2H4 level increased linearly (without diurnal fluctuations) to approximately 1 nL fruit-1 h-1 as the strawberries reached the red-ripe stage. 24 hours after the red fruits began to produce C2H4 again, the CO2 levels increased approximately threefold, indicating a climacteric respiration period (25). In 2012, Hu et al. studied the effects of hydrogen sulphide (H2S) on the post-harvest shelf life and antioxidant metabolism of strawberries. Fumigation with H2S gas released from the H2S donor NaHS prolonged the postharvest shelf life of strawberry fruit in a dose-dependent manner (26). Positive effects of hydrogen gas on the nutrition, texture, and sensory freshness of strawberries have been observed (27).

Zhang J and colleagues used an electronic nose (e-nose), headspace solid-phase microextraction-gas chromatography–mass spectrometry (HS-SPME-GC–MS), and gas chromatography-ion mobility spectrometry (GC-IMS) to study the aroma of freshly squeezed strawberry juice during storage at 4 ± 1°C. By analysing the volatile organic compound (VOC) content, they enhanced our understanding of VOCs and provided a basis for studying the aromatic characteristics of freshly squeezed strawberry juice (28). Granitto et al. employed PTR-MS technology to analyse volatile compounds and applied various data mining techniques to achieve rapid and non-destructive detection of individual strawberries (29). Lu developed a mobile electronic nose to detect strawberry decay using gas sensors. Six metal oxide sensors were selected, and the sensor array was combined with a mobile unit and computer to construct a mobile electronic nose device (30). Pan L and colleagues employed an electronic nose and gas chromatography–mass spectrometry (GC–MS) to detect and classify early post-harvest fruit pathogen infections. In the experiment, strawberries inoculated with grey mould, blue mould, and root mould fungi decayed within 2 d at 5°C. A multilayer perceptron neural network model accurately discriminated between four groups of strawberries with fungal infections with an accuracy rate of 96.6% (8). Xing M and colleagues developed a custom electronic nose system called “Red Face” for detecting the freshness of strawberries during different storage periods. The system consists of six metal oxide semiconductor sensors connected to a data acquisition system and a computer equipped with pattern recognition software (31).

Electronic noses often demonstrate good performance in detecting berry fruits such as white berries and blackberries (32). In 2020, Rao Jingshan, Zhang Yuchen, and colleagues investigated the use of (e-nose) technology to predict the volatile organic compounds (VOCs) in vibration-damaged strawberry fruits during storage on all four sampling days. The best models for the residual prediction deviation values were 2.984 and 5.478. The discrimination model for damaged strawberries also achieved good classification results, with an average correct response rate of 99.24% for both calibration and prediction (33). In 2022, Cao Yang, Zhang Yuchen, and others improved upon previous research. By studying the detection of damaged strawberries through VOCs and establishing and predicting models for the degree of damage and impact time, they demonstrated that mechanical impact caused changes in the VOCs in strawberries and that using electronic nose technology to detect impact-damaged strawberries was feasible (34). In 2022, Al-Dairi Mai et al. conducted research on the postharvest transportation of fresh agriproducts and introduced an experimental method for measuring vibration levels during the transportation process. Factors affecting vibration levels include surface conditions, vehicle speed, vibration duration, vibration direction, and packaging units (35).

## 3. Experiment and equipment

### 3.1. E-nose used in the experiment

**Figure 1:**
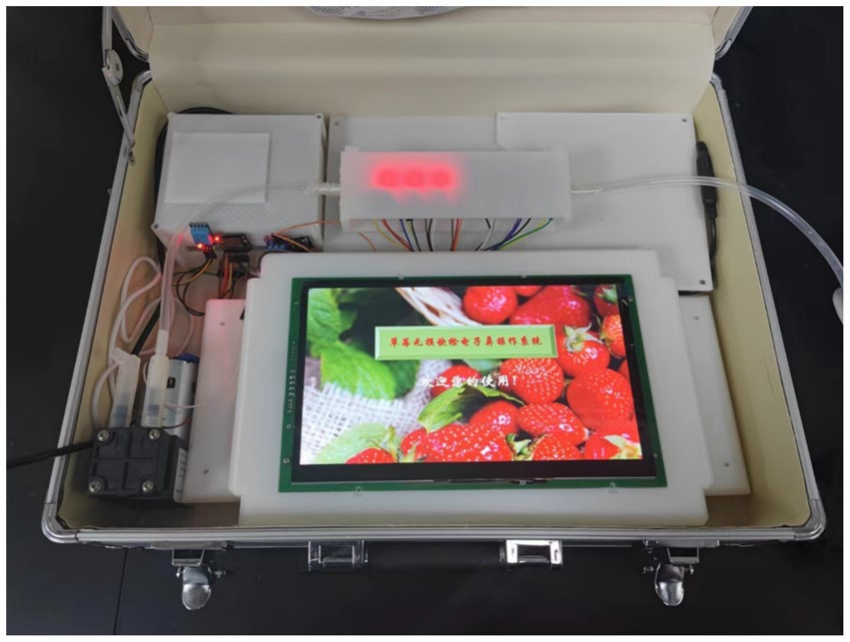
The independently developed e-nose.

As an independent detection device, we must consider the ease of use for operators in addition to the sensor array. We developed a complete set of equipment based on a microcontroller incorporating a 10-inch capacitive touchscreen that integrates the device’s functions for operation and control, including sampling and cleaning switches, experimental data grouping, baseline zeroing, data combination display, column display, and time display. Furthermore, our electronic nose features a multilevel data saving function to ensure data security (36). In an electronic nose system, data stored on a TF card can also be uploaded to a server for backup storage via Wi-Fi. Figure 1 shows the electronic nose used in the experiment.

**Figure 2:**
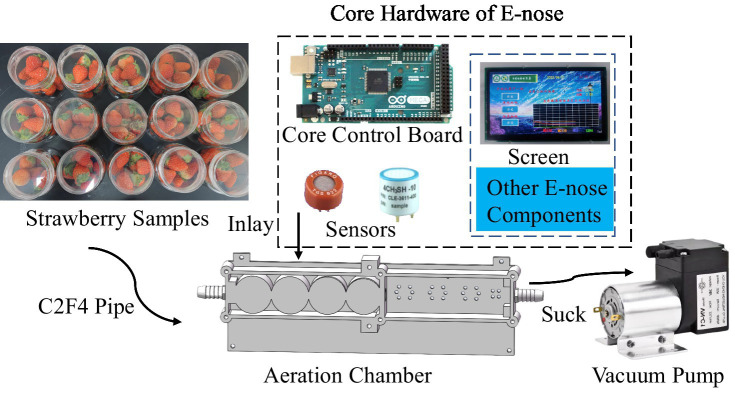
Schematic diagram of the e-nose operation.

Figure 2 depicts the working and structural principles of the electronic nose used in the experiment. The electronic nose was equipped with a 12 W vacuum pump, which, after pulse width modulation (PWM) speed regulation, provided suction to the gas chamber as per demand, thereby drawing the sample gas into the chamber through a hose. The gas chamber contained embedded sensors that transmitted signals to the MCU. The data were calculated and displayed on a 10-inch touchscreen. In addition to the gas information, temperature, humidity, time, and group information were stored in the TF card.

### 3.2. Strawberry sample preparation

**Figure 3:**
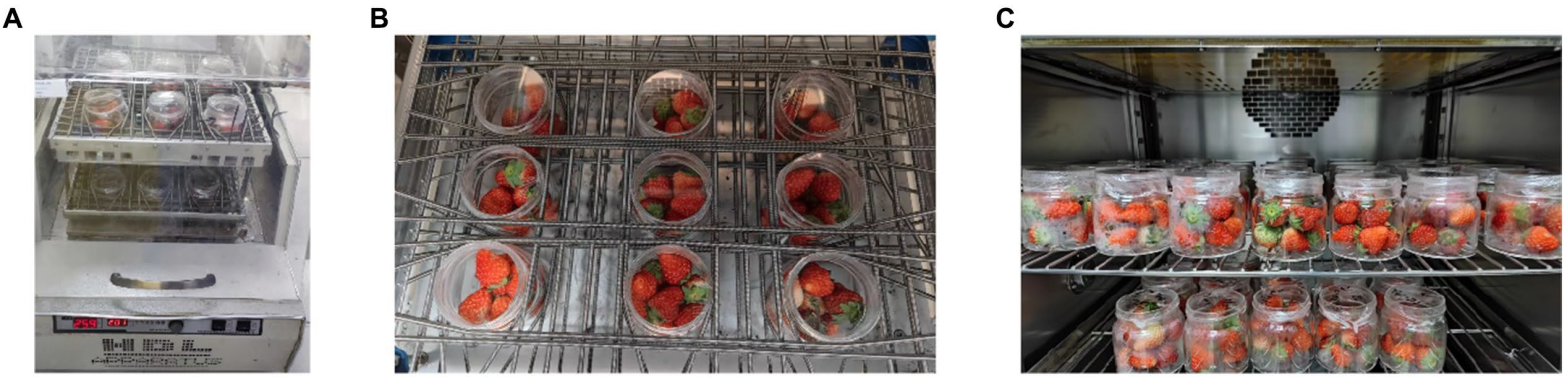
Strawberry pre-processing: **(A)** using H2Q-C air bath shaker for oscillation, **(B)** the distribution of strawberries in the shaker and **(C)** refrigerate at 4°C Celsius.

To obtain strawberries with minor damage, we simulated the damage to freshly harvested strawberries. To simulate the mechanical damage in strawberries, two commonly used methods are freefall (34) and oscillation (33). However, in this particular study, the variety of strawberries investigated (double winter strawberries) was characterised by its small size and abundant fruit quantity, making oscillation the preferred method for simulating mechanical damage to strawberries. To facilitate gas detection, we selected Shuangliu winter strawberries with an average weight of 8.5 g, that were in good and fresh condition. Nine strawberries were placed in a 250 mL wide-mouth bottle and sealed with sealing film. Sealed strawberries were divided into groups to induce varying degrees of minor damage. An H2Q-C air-bath shaker was used to simulate the shaking process. The groups were divided according to the shaking intensity, as shown in Table 1, with 10 parallel samples in each group. After shaking, the samples are placed in a 4.5°C refrigerator for detection (Figure 3).

**Table 1:** Group and oscillation frequency.

Group	Oscillation frequency(r/min)	Time(min)
a	0	0
b	120	60
c	160	60
d	200	60
e	240	60

It is noteworthy that we tested different shakers, and the results varied under different devices, owing to factors such as the degree of fixation of the shaker and equipment resonance. If strawberries continually shift their positions within the bottle, severe damage may occur. However, if strawberries are packed tightly or subjected to high centrifugal force due to rapid rotation, there may be little displacement, resulting in damage caused by compression only. In this experiment, the shaker used at a rotation speed of 240 r/min still led to relatively intense collisions among the strawberries, but did not result in direct damage or loss of the fruit flesh.

### 3.3. Difficulty in sample detection

This experiment was challenging for several reasons. First, the damage to strawberries after shaking was minimal and did not directly damage the flesh. Even when observing with the naked eye, we needed to be close to the samples. After shaking, strawberries may exhibit swelling and darkening of colour, both of which are indicators of damage. Second, unlike previous studies, our experiment involved storing all samples in cold storage. Under refrigeration, strawberries are difficult to oxidise and rot.

These two factors account for the fact that, in the current strawberry harvesting and transportation processes, single-fruit packaging is often employed, road transportation conditions have improved, and the hardness and quality of the fruit have increased, making direct damage less common. Moreover, owing to the short shelf life of strawberries, refrigeration is commonly used for daily storage.

### 3.4. Detection method

Electronic nose collection experiments were conducted on the prepared samples after 24 h, 48 h, 72 h, 96 h, and 120 h of refrigeration. Before starting the experiment, the custom-built electronic nose needs to be preheated for 2–4 h. Once the electrochemical sensors are unbiased and the metal-oxide sensors operate stably, data collection can begin. The collection phase can be divided into ventilation calibration, sampling, and cleaning phases.

#### 3.4.1. Calibration

After basic ventilation calibration, our self-developed electronic nose can respond to sensors in air, perform zero calibration on electrochemical sensors, and output G/G0 for metal oxide sensors.

#### 3.4.2. Sampling

A rigid plastic sampling head was used to quickly puncture the sealing film, and a 12 W vacuum pump gradually pumped the detected gas into the gas chamber.

#### 3.4.3. Cleaning

In comparison to the zeroing method mentioned in Reference (24), we still opt to sacrifice efficiency in favour of sensor accuracy by using a 40 W high-power vacuum pump and waiting for natural zeroing as much as possible. If natural calibration is not possible, wait 10 min after cleaning before recalibration.

After experimental data collection, the obtained sample data had two labels: time and damage groups. The goal was to ensure that the e-nose model built at a later stage could accurately judge strawberry damage under various conditions.

### 3.5. Sensor array selection

Compared to existing electronic noses in the market, we chose to develop our own, tailored to the specific characteristics of the fruit being detected. Pen-3 is a mature commercial electronic nose widely favoured by researchers and is primarily fabricated using ten metal oxide sensors. These metal oxide sensors have broad applicability, a relatively long lifespan, and strong detection sensitivity owing to their cross-sensitivity, although they cannot accurately determine the specific composition of the reacted substances.

**Figure 4:**
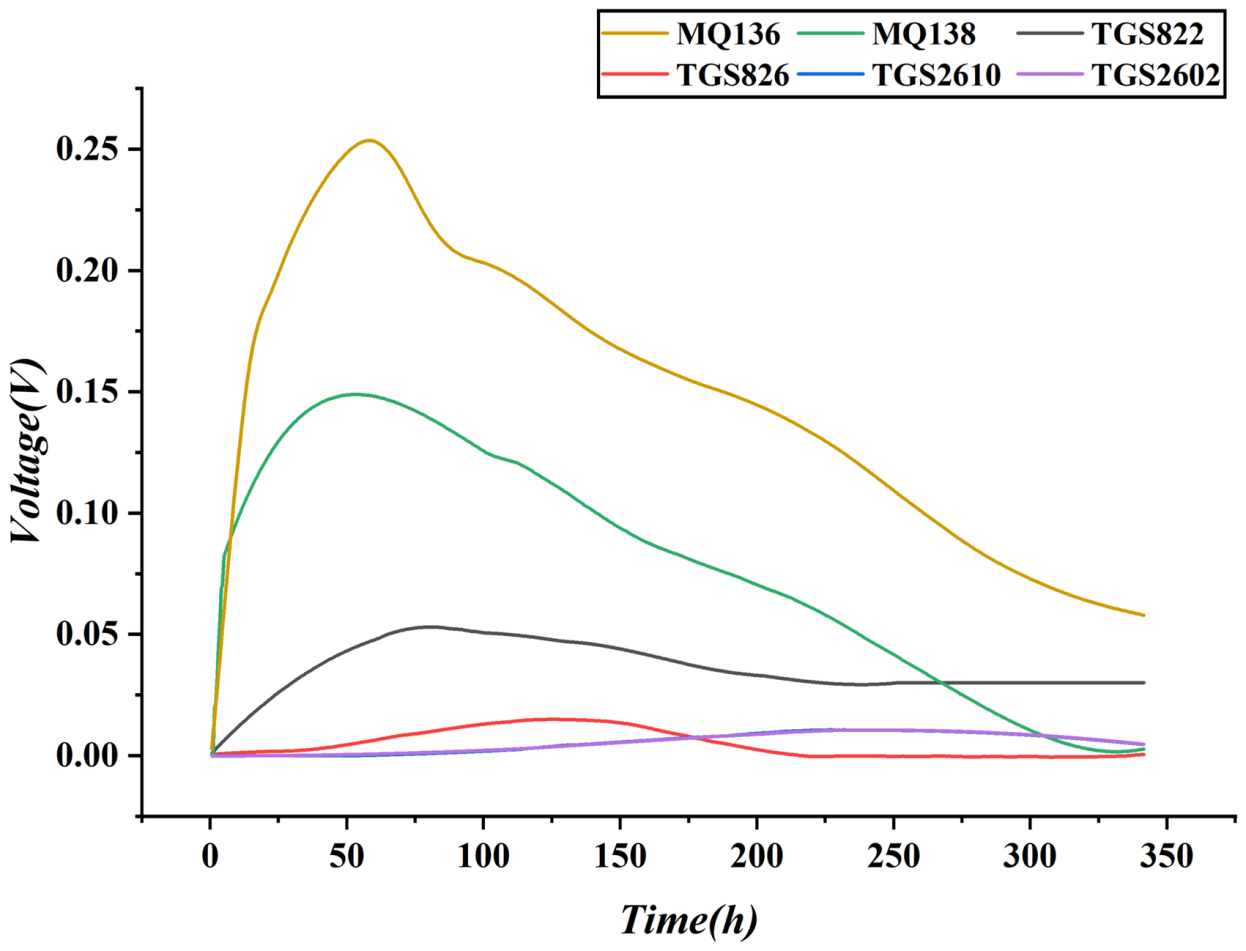
Comparison of experimental results for metal oxide sensors.

The method of selecting metal oxide sensors to detect the corresponding substances was in accordance with Reference (31); however, the models of the selected sensors were not the same. Preliminary tests were conducted on other batches of strawberries under the same experimental conditions. We applied the Savitzky–Golay method to denoise the data and plotted the response curves of the sensor modules over time (Figure 4) to select relatively sensitive metal-oxide sensors. It can be observed that the curves of TGS2602 and TGS2610 overlap; therefore, only one curve needs to be retained. The response of TGS826 was not obvious, whereas MQ136, MQ138, and MQ822 had relatively higher contributions. To ensure sufficient gas chamber space and increase the contact area between the unit gas and the sensor, TGS826 and TGS2602 were removed, leaving only MQ136, MQ138, TGS826, and TGS2602.

Electrochemical sensors have a more precise range and can accurately determine the chemical changes between the sensor and the reacted substances using millivolt-level voltages, thus obtaining precise reaction changes. However, electrochemical sensors mainly measure specific inorganic substances with a relatively high specificity. In existing strawberry research, the detection of substances, such as ethylene, sulphides, and alcohols, is often required. Therefore, we prioritised the selection of electrochemical sensors that can detect these types of substances.

**Figure 5:**
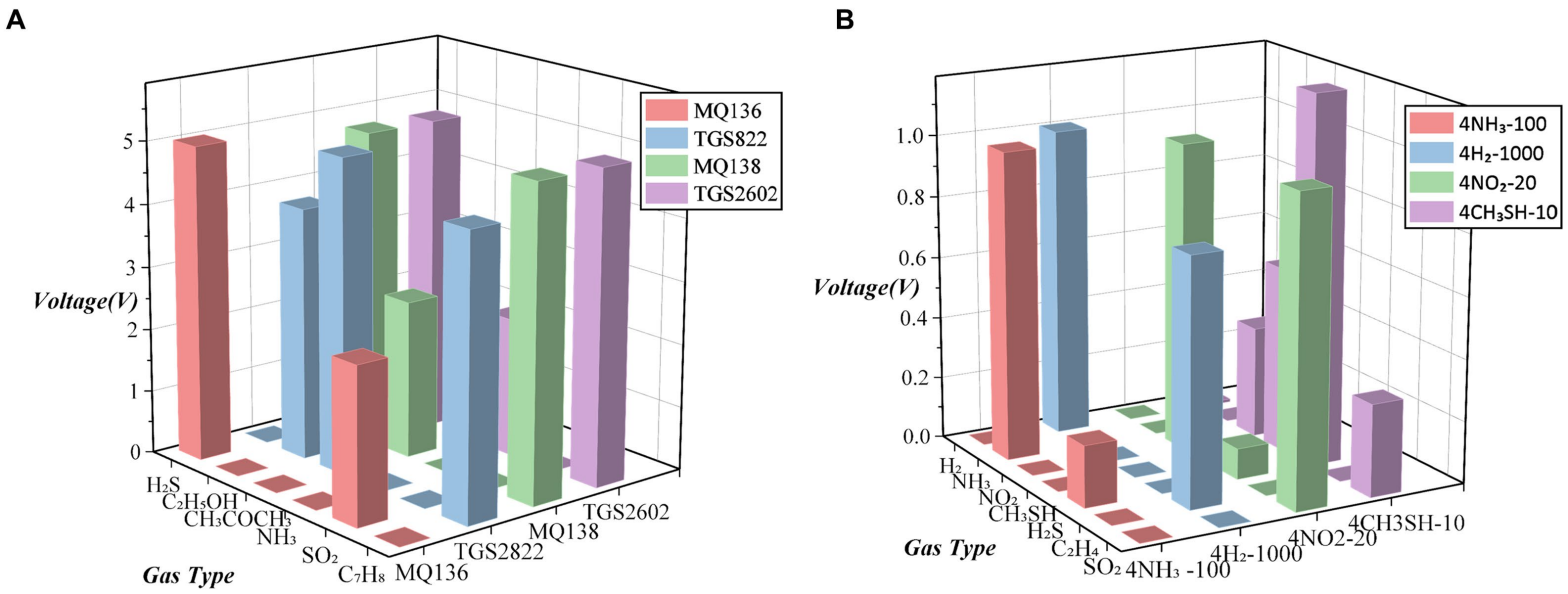
Sensitive response characteristic substances of 8 types of sensors: **(A)** sensitive substances for metal oxide sensors and **(B)** sensitive substances for electrochemical sensors.

As a result, we chose the metal oxide sensors TGS822, TGS2602, MQ136, and MQ138 and the electrochemical sensors 4CH_3_SH-10, 4NO_2_-20, 4NH_3_-100, and 4H_2_-1,000. Based on the voltage change characteristics of the two types of sensor modules, we plotted Figure 5, which shows their sensitivity to various substances under known conditions. A value of 0 indicated no reaction or an unclear reaction. The sensitivity of these sensors to various substances was sufficient to detect the changes caused by strawberry volatiles.

Electrochemical sensors are more precise in their measurement range, and by utilising their millivolt-level voltage, they can accurately determine the chemical changes between the sensor and the substances being reacted, thereby obtaining precise reaction changes. However, electrochemical sensors are mainly used to measure a few inorganic substances and have a relatively high specificity for detection. In existing strawberry research, the detection of substances, such as ethylene, sulphides, and alcohols, is often required. Therefore, we prioritised the selection of electrochemical sensors that can detect these types of substances. Metal-oxide sensors have high repeatability and stability, allowing them to operate stably over long periods of time. In addition, they exhibit different characteristics in response to different gases, which enables them to distinguish and identify different gases.

### 3.6. Experimental phenomenon

**Figure 6:**
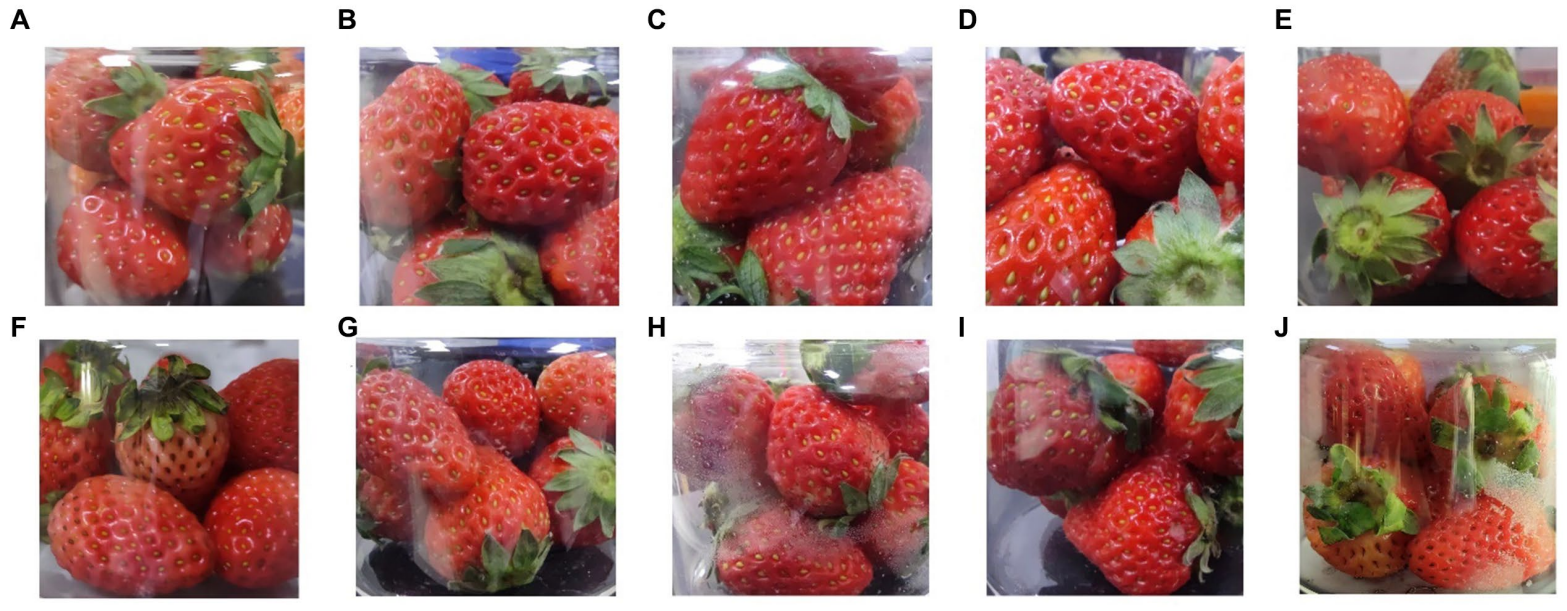
The visual observations of strawberries from the different groups. Photographs **(A–E)** were taken immediately after oscillation, when the strawberries were fresh, and photographs **(F–J)** were taken after 72 h of cold storage. Photographs **(A–E)** and **(F–J)** both correspond to the groups **(A–E)** described in Table 1.

Strawberries exhibited varying levels of damage depending on the applied oscillation frequency. Group A represented undamaged strawberries, whereas group B consisted of samples obtained at a rotation speed of 120 rpm. The rotation speed increased progressively throughout the groups, with group E containing samples obtained at 240 rpm (Table 1). To ensure a proportional relationship between the rotation speed and the degree of damage, it is crucial that strawberries collide and move, even at the highest rotation speed. Strawberries with abnormal oscillations were removed by visual inspection. The visual distinctions among different groups can be discerned by examining the captured images of strawberries (Figure 6).

Group A contained undamaged strawberries. They are fresh, glossy, vibrant, and well formed, with a faintly sweet fragrance. Even after cold storage, they remained intact, although some of their gloss was lost.

In group B, strawberries exhibited slight surface wrinkling due to mild oscillation. Their scent was similar to that of group A. After a few days of cold storage, some strawberries in Group B showed a slight darkening of colour, but those with uneven collisions maintained their freshness.

Group C strawberries experienced an increase in wrinkle depth, and their edges became less defined. Their scent was similar to that of Group A, but slightly stronger. After several days of cold storage, the fruit loses moisture and the dark red colour becomes more pronounced.

In group D, the collision frequency reached a high-intensity rotation speed of 200 rpm, resulting in several strawberries with large areas of wrinkling and a dark red colour. They appeared soft and mushy, with a sweeter scent. After cold storage, the collision areas darken further, and dehydration issues arise, accompanied by a stimulating sweet aroma.

Finally, group E strawberries could not maintain their original angular shape after oscillation and gradually became smooth and round. Generally, they are soft and mushy. After a few days of storage, they developed a rich sweet scent that grew increasingly intense.

## 4. Results and analysis

### 4.1. Electronic nose detection data

**Figure 7:**
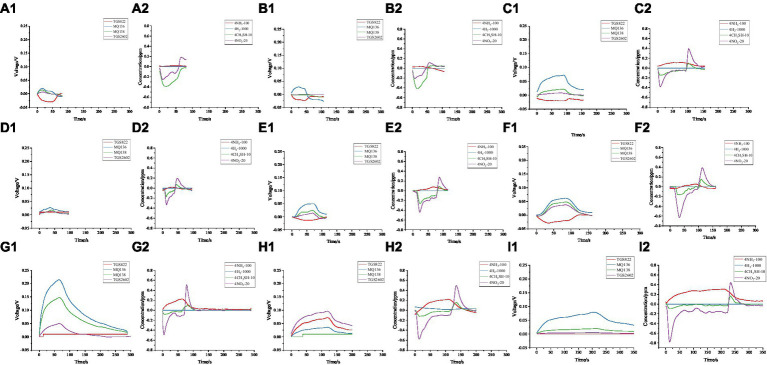
Comparison of electronic nose responses for individual samples at different experimental stages: **(A1)**, **(B1)**, and **(C1)** represent the voltage changes of the metal oxide sensor module for group A samples at 24 h, 72 h, and 120 h, respectively. **(A2)**, **(B2)**, and **(C2)** represent the changes in gas concentration detected by the electrochemical sensor module for group A samples at 24 h, 72 h, and 120 h, respectively. Similarly, **(D1)**, **(E1)**, and **(F1)** represent the voltage changes of the metal oxide sensor module for group C samples at 24 h, 72 h, and 120 h, while **(D2)**, **(E2)**, and **(F2)** represent the changes in gas concentration detected by the electrochemical sensor module for group C samples at 24 h, 72 h, and 120 h. **(G1)**, **(H1)**, and **(I1)** represent the voltage changes of the metal oxide sensor module for group E samples at 24 h, 72 h, and 120 h, while **(G2)**, **(H2)**, and **(I2)** represent the changes in gas concentration detected by the electrochemical sensor module for group E samples at 24 h, 72 h, and 120 h.

To clearly show the changes in the sample gas, we provide an example from the experimental data (Figure 7). As the degree of damage changed over time, the sensor signals exhibited different variations. One noticeable observation was that the required detection time varied. On average, group E samples took longer to detect than group A and C samples. This may be because, when gas exchange occurs, the gas diffuses and intermingles. When the gas concentration is high, it takes longer for the gas to fully diffuse in the gas chamber; consequently, the rise and fall of the gas concentration also require more time. Different sensors exhibit varying sensitivities at different response stages. To further analyse this, we calculated all data.

**Figure 8:**
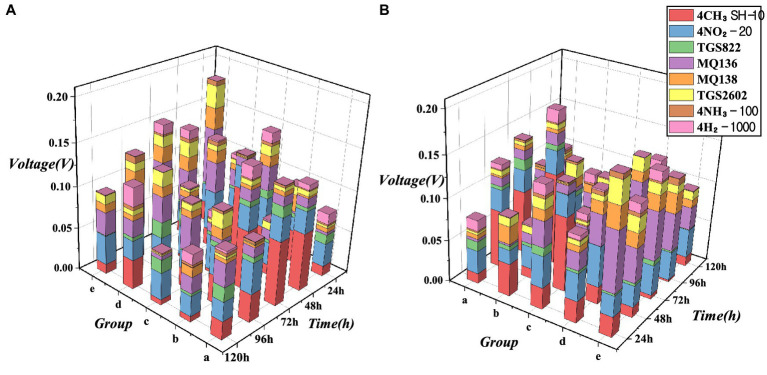
Voltage proportion of the 8 sensors under various experimental conditions: **(A)** viewpoint 1st and **(B)** viewpoint 2nd.

Based on the experimental results, the response ratios of the sensors were visualised using eight 3D stacked bar charts of the voltage ratios of the metal oxide sensors (Figure 8). Calculations were performed on all the raw data from the experiments. The charts depict the voltage values (V) of each sensor during the entire response phase for different groups (groups) and times (h). These visualisations demonstrate the proportion of each sensor response under various experimental conditions. First, because the voltage range of metal oxide sensors differs from that of electrochemical sensors, we scaled the electrochemical sensor data to match the voltage range of the metal oxide sensors for comparison. Next, we calculated the mean values for each sensor during the entire reaction cycle (sampling, rising, peaking, and cleaning). This approach, which incorporates all the data, offers greater stability and versatility than the use of maximum or minimum absolute values. It can offset fluctuating electrical signals near 0, and conversely, it can include stable values throughout the entire reaction process. The following information can be obtained from the chart.

1. The chart displays only the activity of the sensors and does not reflect their contributions to the results. The higher the proportion of the sensor, the higher is its activity, which may also be influenced by noise. However, for different orders of magnitude, a sensor with a larger proportion is more likely to be sensitive to the reactants.
2. The mean voltage represented by the y-axis does not reveal the voltage pattern, because the data used were from the entire detection process. The peak and cleaning times varied under different experimental conditions (e.g. the detection time for group E was several times that for group A). The time required to reach the peak and the time required to complete the cleaning process differed for the samples under different conditions. A low voltage might indicate a small reaction; however, in most cases, this is due to prolonged cleaning times, which lowers the average voltage value after taking the mean. Therefore, only the proportions of various sensors can be compared.
3. The sensors with the lowest proportions were 4H_2_-1000 and 4NH_3_-100. Possible reasons for their low responses are either an overly large measurement range or a small amount of reactive gases.
4. Sensors TGS2602 and MQ138 have a slightly smaller proportion than MQ136, which is consistent with experimental expectations.
5. The sensors with the highest proportions were 4CH_3_SH-10, 4NO_2_-20, MQ136, and TGS822. For electrochemical sensors 4CH_3_SH-10 and 4NO_2_-20, their small measurement range allow them to capture reaction states more sensitively within their range. For MQ136 and TGS822, this was because of the presence of sensitive reactants corresponding to them.
6. The response level (also known as the activity level) of each sensor may vary under different conditions. For example, the red 4CH_3_SH-10 sensor may become less active than the other sensors as the damage level increases. The MQ136 sensor's activity level may also increase as the time interval and damage level increase. However, some sensors do not exhibit a clear pattern, and may require other statistical or processing methods for analysis.

### 4.2. Use PCA to perform feature extraction on the raw data

Dimensionality reduction of the collected full-process data using PCA: The sample data obtained from the electronic nose is a time series, which is continuous data that change over time. Different sensor data were generated at each time point, and the data for each period constituted a sample. By employing principal component analysis (PCA) for dimensionality reduction, the original eigenvalues became matrices. In time series data, each sample typically contains data from multiple time points. We consider each time point as a feature and each sample as a vector, allowing us to convert the time-series data into a matrix. Subsequently, we can perform PCA on this matrix to obtain low-dimensional data features.

#### 4.2.1. Data for different damage groups

**Figure 9:**
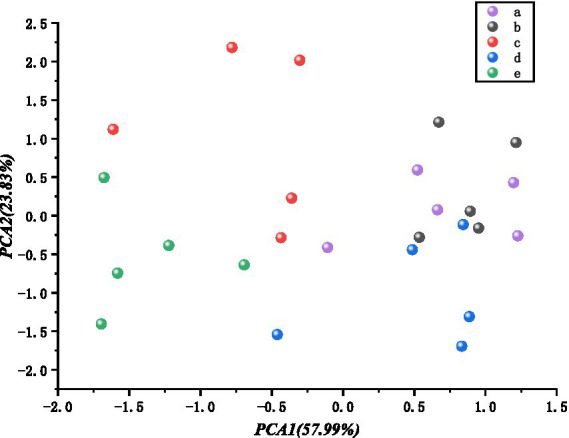
Comparison of images before and after cold storage for different groups.

This set of data simultaneously shows the electronic nose response data for different damage groups A–E at the same time. PCA dimensionality reduction is applied to address the issue of determining the degree of damage. PCA1 and PCA2 account for more than 81% of the total, and the first three principal components account for 91%. As shown in Figure 9, the first two principal components accounted for 81% of the explainable variance, and points within the same group were clustered together. However, the boundaries were blurred and there was some overlap between groups A and B. This could be due to the low level of damage in Group B, which was not significantly different from the undamaged state, making it difficult to distinguish.

#### 4.2.2. Data for different detection times

**Figure 10:**
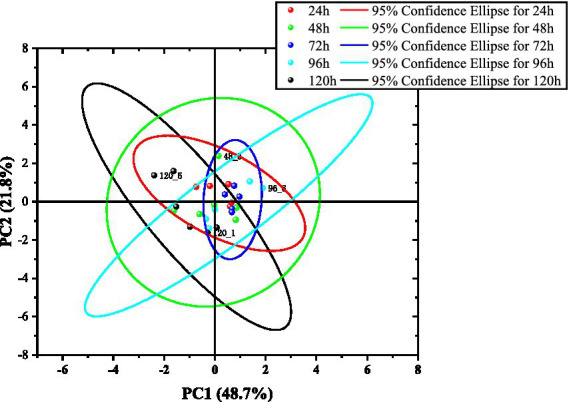
Comparison of images before and after cold storage for different groups.

Subsequently, calculations were carried out for determining time periods, using strawberry samples with the same degree of damage, comparing their electronic nose voltage responses between 24 h and 120 h of storage at 4°C. PCA1 and PCA2 account for more than 70% of the total, with the first four principal components exceeding 91%. Figure 10 shows that the confidence ellipses have multiple orientations, indicating different trends in different principal component directions and large variances in these directions. The confidence ellipse shapes and sizes vary. Although the data points are relatively concentrated, possibly owing to the small mean differences between multiple groups, the variances can be clearly distinguished, and there is a clustering trend for points of the same type.

Because the damage changes among the five groups of strawberries were not significant and the storage over time was at a low temperature, it was difficult to distinguish the obtained data. The introduction of data collection throughout the entire process (zero baseline, response emergence, peak, stability, and cleaning) may result in various situations that are difficult to interpret and classify, particularly the uncertainty of the cleaning time. In addition, the lack of designed weight values causes less important times and critical judgment points to have the same weight, thereby reducing their characteristic representations.

Although the PCA chart does not directly distinguish between groups, the size, direction, and aggregation of confidence ellipses and scatter points display a rich range of possibilities, indicating potential value for classification.

### 4.3. Use eigenvalue extraction methods to perform feature extraction on the raw data

Using eigenvalue extraction methods: The advantage of the electronic nose feature extraction is its flexibility in choosing different eigenvalues according to the requirements of the application scenario. It can be combined with domain knowledge and experience for selection and is convenient for interpreting practical meanings. In EN data processing of the electronic nose, it is necessary to extract effective eigenvalues from raw data for pattern recognition and classification. In this study, we propose nine effective eigenvalues: the maximum value, minimum value, mean value, Variance, Differential mean, integral mean, start-up differential value, average slope near the extreme value, and count of data points greater than the absolute mean value (abbreviated as count). Owing to the uncertainty in the detection time, the differential and integral means are more practically meaningful. The integral mean was calculated using a trapezoidal formula. The average slope near the extreme value has a peak shape. The larger the value, the sharper the peak, and the smaller the value, the smaller the peak. Moreover, because a stronger odour can result in a longer cleaning time, which can lower the mean value, the count is a good indicator of samples with strong volatility.

### 4.4. Prediction model

The experimental data come from two sources: one is the PCA values obtained through dimensionality reduction of the time-series PCA, where their sum is greater than 95%, and the other is the dimensionality reduction using eigenvalues. The calculation method for the eigenvalues was introduced in the previous section, and nine eigenvalues were selected. MQ138 and TGS2602, which exhibited response curves similar to those of MQ136, were removed to reduce the probability of overfitting. Among the remaining six sensors, 4H2-1,000 and 4NH3-100 had minimal impact owing to their larger measurement ranges compared to the measured gas content. In clean air, the voltage fluctuations are of the same order of magnitude as those in the sensitive gas; therefore, most eigenvalues are not applicable. Only the means and variances of the two sensors were retained to avoid adverse effects. Finally, four of the six sensors retained nine eigenvalues and two sensors retained two eigenvalues, resulting in a total of 40 eigenvalues per sample. A comparison of the two methods is presented in Table 2.

**Table 2:** Comparison of classification accuracy between the two dimensionality reduction methods.

Problem classification	Dimensionality reduction method	Classification methods	Cross-validation	Accuracy
Time classification	Feature extraction	Subspace ensemble	5	0.88
Time classification	PCA	Subspace ensemble	5	0.6
Damage classification	Feature extraction	Subspace ensemble	5	0.81
Damage classification	PCA	SVM	5	0.38

The dimensionality-reduced experimental data from both methods were imported into classifiers for classification. The classifiers mainly employ the LDA (37), SVM (38), KNN (39), naïve Bayes (40), and Subspace Discriminant (41) methods, among which the Subspace Discriminant is an ensemble classifier. The accuracy of the directed extraction method using eigenvalues was significantly higher than that of the PCA method applied to all the data in the time series. Moreover, performing PCA on the extracted 40 eigenvalues did not improve the accuracy.

For the damage problem, the ensemble classifier Subspace Discriminant performed the best, reaching an accuracy of 0.89, whereas the model for the timing problem achieved an accuracy of 0.81. This enabled a relatively clear classification of the five data categories. Table 3 presents the effects of the other classifiers, with SVM, KNN, and Linear Discriminant Analysis having relatively good classification results.

**Table 3:** The accuracy of the first four models for the two classification problems.

Problem classification	Classification methods	Accuracy
Time classification	Subspace discriminant	0.89
Time classification	SVM	0.81
Time classification	Fine KNN	0.77
Time classification	Naive Bayes	0.77
Damage classification	Subspace discriminant	0.82
Damage classification	LDA	0.78
Damage classification	Medium Gaussian SVM	0.78
Damage classification	Weighted KNN	0.74

### 4.5. Model optimization

At the current stage of research, there are still some limitations. The sensor selection can be further optimised. By comparing and verifying a greater variety of sensor models, the best sensor types can be selected, thereby enhancing the recognition capabilities of the electronic nose from a hardware perspective. There is also room for improvement in data extraction. One possible approach is to process the data only prior to the peak, thus avoiding the negative impact caused by the uncertainty of stabilisation and cleaning times. The selection of feature values should also involve comparing a wider range of extraction methods to further improve accuracy.

From Table 4, it can be concluded that by using Grid Search optimization, the accuracy of SVM was improved from the original 0.80 to 0.84 using Grid Search SVM (GS-SVM), and the PCA-reduced data increased from the original 0.38 to 0.46. As shown in Table 5, using the GS-SVM method, the accuracy increased from 0.71 to 0.84%; however, it was not as high as that of the subspace discriminant model, which was 0.89. For the original PCA-processed data, the accuracy increased from 0.40 to 0.58. The performance of the random search-optimised SVM (RS-SVM) was not as good as that of the GS-SVM. The final experimental results for damage and time discrimination accuracy are shown in Table 6, where the subspace discriminant model achieved 0.89 for a time classification problem and 0.84 for a damage degree classification problem.

**Table 4:** Comparison of classification accuracy results for the time-based problem.

Dimensionality reduction method	Optimization method	Accuracy	Cross-validation
Feature extraction	None	0.80	5
PCA	None	0.38	5
Feature extraction	Grid search	0.84	5
Feature extraction	Random search	0.84	5
PCA	Grid search	0.46	5
PCA	Random search	0.34	5

**Table 5:** Comparison of classification accuracy results for the damage-based problem.

Dimensionality reduction method	Optimization method	Accuracy	Cross-validation
Feature extraction	None	0.71	5
PCA	None	0.40	5
Feature extraction	Grid search	0.84	5
Feature extraction	Random search	0.84	5
PCA	Grid search	0.58	5
PCA	Random search	0.42	5

**Table 6:** Final accuracy.

Problem classification	Dimensionality reduction method	Accuracy	Classification methods	Cross-validation
Time classification	Feature extraction	0.89	Subspace ensemble	5
Time classification	PCA	0.6	Subspace ensemble	5
Damage classification	Feature extraction	0.84	GS-SVM	5
Damage classification	PCA	0.58	GS-SVM	5

## 5. Discussion

### 5.1. Limitations and future improvements

At the current stage of research, there are still some limitations. The sensor selection can be further optimised. By comparing and verifying a greater variety of sensor models, the best sensor types can be selected, thereby enhancing the recognition capabilities of the electronic nose from a hardware perspective. There is also room for improvement in data extraction. One possible approach is to process the data only prior to the peak, thus avoiding the negative impact caused by the uncertainty of stabilisation and cleaning times. The selection of feature values should also involve comparing a wider range of extraction methods to further improve accuracy.

Moreover, it is essential to further optimise the sample space, acquire more valid data, and enhance the robustness of detection. As research progresses, these areas of improvement can be addressed, leading to a more refined and effective non-destructive electronic nose technology for strawberries.

### 5.2. Applications and significance

The development prospects for non-destructive strawberry detection technologies are broad. Food safety regulatory agencies can use this technology to spot-check strawberries to ensure consumer food safety and health. Non-destructive electronic nose technology can be applied during the production and processing of strawberries to monitor and control their quality and freshness. Production and processing companies can use this technology to screen raw strawberries and ensure product quality and safety.

Non-destructive electronic nose technology for strawberries will continue to be developed and improved, and its application scope will become more extensive. Simultaneously, electronic nose technology will continue to innovate, thereby improving its detection accuracy and sensitivity, and better serving people’s lives and health.

## 6. Conclusion

In this study, an electronic nose was developed to detect minor mechanical damage to strawberries. A shaker with a range of 120–240 r/min was used to simulate minor mechanical damage in strawberries, which were stored at 4°C for 120 h under low-temperature refrigeration. An array of sensors specifically designed to detect volatile gases emitted by strawberries was employed in the experiments. Among the electrochemical sensors, 4CH_3_SH-10 and 4NO_2_-20 exhibit the best detection responses. Among the metal oxide sensors, MQ136 and TGS822 demonstrated the highest responsiveness. Various feature extraction methods were applied to effectively capture the characteristics of strawberry data, and the electronic nose achieved an accuracy rate of over 0.8 for the detection of minor mechanical damage in strawberries. The multiclassification accuracy for determining the timing of strawberry damage was 0.84, and that for determining the degree of strawberry damage was 0.89. The experimental results indicated that the device could effectively differentiate the degree of mechanical damage in grouped strawberries and roughly estimate the time at which the damage occurred.

## Data availability statement

The original contributions presented in the study are included in the article/Supplementary material, further inquiries can be directed to the corresponding author.

## Author contributions

YQ: equipment design and manufacturing, formal analysis, experiment, data processing and analysis, designing and creating charts and graphs, writing – original draft, and writing – review and editing. WJ: conceptualization, methodology, formal analysis, supervision, and writing – review and editing. XS: equipment design and manufacturing, and experiment. HL: experiment, and writing – review and editing. All authors contributed to the article and approved the submitted version.

## Funding

This study was funded by the Key Research and Development Projects of Hebei Province (21375501D), Innovation and Capacity Building Project of Beijing Academy of Agriculture and Forestry Sciences (KJCX20200213) and Innovative Entrepreneurial Team Project of Anhui Institute of Innovation for Industrial Technology (LAY-2022-CXGB-006) and National Natural Science Foundation of China (No. 31872094) and Capacity Building Project of Beijing Academy of Agriculture and Forestry Sciences (KJCX20230309).

## Conflict of interest

The authors declare that the research was conducted in the absence of any commercial or financial relationships that could be construed as a potential conflict of interest.

## Publisher’s note

All claims expressed in this article are solely those of the authors and do not necessarily represent those of their affiliated organizations, or those of the publisher, the editors and the reviewers. Any product that may be evaluated in this article, or claim that may be made by its manufacturer, is not guaranteed or endorsed by the publisher.
